# Prediction of lymphovascular space invasion in endometrial cancer using the 55-gene signature selected by DNA microarray analysis

**DOI:** 10.1371/journal.pone.0223178

**Published:** 2019-09-26

**Authors:** Takafumi Watanabe, Reiko Honma, Manabu Kojima, Shinji Nomura, Shigenori Furukawa, Shu Soeda, Shinya Watanabe, Keiya Fujimori

**Affiliations:** 1 Department of Obstetrics and Gynecology, Fukushima Medical University School of Medicine, Fukushima, Japan; 2 Nippon Gene Co., Ltd., Chiyoda, Tokyo, Japan; 3 Medical-Industrial Translational Research Center, Fukushima Medical University School of Medicine, Fukushima, Japan; Chang Gung Memorial Hospital at Linkou, TAIWAN

## Abstract

Lymphovascular space invasion (LVSI) is considered to be the beginning of lymphogenous and hematogenous metastases. It is strongly related to dissemination, and therefore could be a valuable predictive sign of lymph node metastases and distant spread. Recently, the presence of LVSI in endometrial cancer (EC) has been shown to be an independent prognostic factor. The preoperative diagnosis of LVSI by pathological examination is difficult and LVSI is detected after surgery. The aim of the current study was to explore candidate genes as potential diagnostic biomarkers and determine whether they are predictors of LVSI in patients with EC. A total of 88 surgical specimens obtained from EC patients who had undergone surgical resection at Fukushima Medical University Hospital between 2010 and 2015 were analyzed using DNA microarray. LVSI was significantly associated with poor prognostic factors in EC such as higher tumor grade, lymph node metastasis, deep myometrium invasion, advanced stage and recurrence. Fifty-five candidate genes were significantly differentially expressed between 26 LVSI-positive and 62 LVSI-negative samples. All 88 samples were divided into two groups according to hierarchical clustering of 55 genes. Regarding diagnostic accuracy, sensitivity and negative predictive value were both high (92% and 95%, respectively); further, specificity and positive predictive value were both moderate (63% and 71%, respectively). Our data suggests that the 55-gene signature could contribute to predicting LVSI in EC, and provide clinically important information for better management. The molecular signatures of 55 genes may be also useful for understanding the underlying mechanism of LVSI.

## Introduction

Endometrial cancer (EC) is the most common malignancy of the female genital tract in the world, and the sixth most commonly occurring cancer in women worldwide [1]. EC often produces symptoms, such as postmenopausal or abnormal genital bleeding, at a relatively early stage, so the disease is generally diagnosed early. This is evidenced by the finding that more than 75% of EC cases are stage I at the time of diagnosis, and the 5-year overall survival (OS) rate for women with early-stage EC exceeds 80% [2]. However, depending on pathologic factors, up to 30% of reported early stage patients had pelvic regional recurrence disease, and 5–6% had distant recurrence [3]. There are a number of known prognostic factors for EC as defined by the International Federation of Obstetrics and Gynecology (FIGO), including stage, tumor grade, histological type, and deep myometrial invasion [4].

Lymphovascular space invasion (LVSI) is a crucial first step of tumor metastasis and is defined as the invasion of tumor cells in lymphatic and/or blood vessels. Although FIGO 2009 staging classification does not include LVSI as a prognostic factor for EC, LVSI has recently been shown to be an independent prognostic factor in several studies [5, 6]. Furthermore, multivariate analysis, adjusted for several pathological parameters known to affect clinical outcome, has shown that LVSI is an independent predictor for lymph node and distant metastases [7, 8]. As for pathological LVSI diagnosis, interobserver variability in the evaluation of LVSI cannot be overlooked, because of the difficulties in comprehending lymphatic channels and blood vessels using standard hematoxylin-and-eosin staining alone [9]. An accurate indicator, instead of pathological examination, is necessary for LVSI diagnosis in EC patients.

The aim of the current study was to explore candidate genes as potential diagnostic biomarkers for LVSI in EC by comprehensive gene expression analysis using DNA microarray. We also present novel data of a gene expression profile related to LVSI.

## Materials & methods

### Tissue specimens

A total of 88 surgical specimens were obtained from EC patients who had undergone surgical resection at Fukushima Medical University Hospital between 2010 and 2015. Pathological diagnosis for histologic cell type, tumor differentiation, myometrium invasion, lymph node metastasis and staging of disease was performed. Staging was determined according to the FIGO classification (2008). Written informed consent was obtained from all patients, and the study design was approved by the ethics committee of Fukushima Medical University (approval number: 1953).

The Mann-Whitney U test was used to compare median age and body mass index (BMI) of patients with and without LVSI. The Fisher exact test was used to compare stage, histology, grade, myometrium invasion and lymph node metastasis between patients with and without LVSI. The survival curve was generated by using the Kaplan Meier technique and differences between these curves were tested by the log-rank test. All statistical analyses were performed using SPSS version 20.0 software (SPSS, Inc., Chicago, IL, USA), and statistical significance was defined as *P*<0.05.

### Comprehensive gene expression analysis

Frozen specimens were processed for total RNA extraction using Isogen (Nippon Gene Co., Ltd., Tokyo, Japan) and for poly(A)+RNA purification using MicroPoly(A) Purist kit (Ambion, Austin, TX, USA). The DNA microarray that used for poly(A)+RNA was named system 1; a set of synthetic polynucleotides (80-mers) representing 31,797 species of human transcript sequences was printed on a glass slide using a custom arrayer. The DNA microarray that used for total RNA was named system 2; a set of synthetic polynucleotides (80-mers) representing 14,400 species of human transcript sequences was printed on a glass slide using a custom arrayer. For RNA of the samples, SuperScript II (Invitrogen Life Technologies, Carlsbad, CA, USA) and Cyanine 5-dUTP (Perkin-Elmer Inc., Boston, MA, USA) were used to synthesize labeled cDNA from 2 μg of poly(A)+RNA in system 1 and 5 μg of total RNA in system 2. Using the same method for the human common reference RNA, Cyanine 3-dUTP (Perkin-Elmer Inc.) was used to synthesize labeled cDNA from 2 μg of Human Universal Reference RNA Type I (MicroDiagnostic, Tokyo, Japan) in system 1 and 5 μg of Human Universal Reference RNA Type II (MicroDiagnostic) in system 2.

Hybridization was performed with a Labeling and Hybridization kit (MicroDiagnostic). Signals were measured using a GenePix 4000B Scanner (Axon Instruments, Inc., Union City, CA, USA) and then processed into the primary expression ratios of the cyanine 5 intensity of each specimen to the cyanine 3 intensity of the Human Universal Reference RNA. Each ratio was normalized using GenePix Pro 3.0 software (Axon Instruments, Inc.,). The primary expression ratios were converted into log2 values, which were designated as log ratios or converted value. Date were processed using Microsoft Excel software (Microsoft, Bellevue, WA, USA) and MDI gene expression analysis software package (MicroDiagnostic) [10, 11].

### Hierarchical clustering

To detect genes that were associated with LVSI, we analyzed DNA microarray data using log ratios. Firstly, genes with fluorescence intensity below the detection limit in seven or more of the 26 LVSI-positive samples and/or in sixteen or more of the 62 LVSI-negative samples were excluded. Secondly, we calculated the means of the converted values of 26 LVSI-positive samples and 62 LVSI-negative samples for each remaining gene, then, the genes that met the following requirement were selected: the mean of 26 LVSI-positive samples–the mean of 62 LVSI-negative samples ≥1. Finally, a Student’s t-test was used to compare the converted value between the LSVI-positive samples and the LSVI-negative samples. The genes with a p-value of <0.005 were selected. Hierarchical clustering was analyzed using the Euclidean distance and performed by EpressionView Pro software (MicroDiagnostic).

Sensitivity, specificity, positive predictive value (PPV), and negative predictive value (NPV) of the hierarchical clustering using the gene set were calculated using SPSS version 20.0 software (SPSS, Inc., Chicago, IL, USA).

### Gene Ontology

Gene Ontology (GO) enrichment analysis was performed using the Panther (http://pantherdb.org), which uses a selected set of terms from the GO for classifications by molecular function, biological process and cellular component. The classification process was extensively explained by Mi *et al*. [12].

## Results

### Clinicopathological characteristics

The clinical data of the 88 patients who underwent surgery for EC is summarized in Table 1. The numbers of LVSI-positive and LVSI-negative patients were 26 and 62 respectively. The total number of 80 patients had lymph node dissection and the remaining eight patients did not undergo pathological evaluation with a lymphadenectomy. The populations of the patients in the LVSI-positive and negative groups were significantly different in terms of BMI stage, tumor grade, deep myometrial invasion, lymph node metastasis, and recurrence (Table 1). However, there was no significant difference in age or tumor histology between the groups (Table 1). LVSI-positive was significantly associated with worse OS (S1 Fig)

**Table 1:** Clinicopathological characteristics of LVSI positive and negative groups.

Characteristic	LVSI positive (n = 26)	LVSI negative (n = 62)	*P*
Age, mean±SD	63±12.1	57±13.1	0.05
BMI, mean±SD	22.7±4.7	27.0±9.3	0.03
Stage			
I/II	9	50	
III/IV	17	12	<0.001
Histology			
Endometrioid	22	56	0.44
Nonendometrioid	4	6	
Grade			
1	6	48	
2/3	20	14	<0.001
Myometrial invasion			
<50%	20	10	
>50%	6	52	<0.001
Lymph node metastasis			
Yes	10	1	
No	9	60	<0.001

### Identification of genes to differentiate LVSI

**Fig 1:**
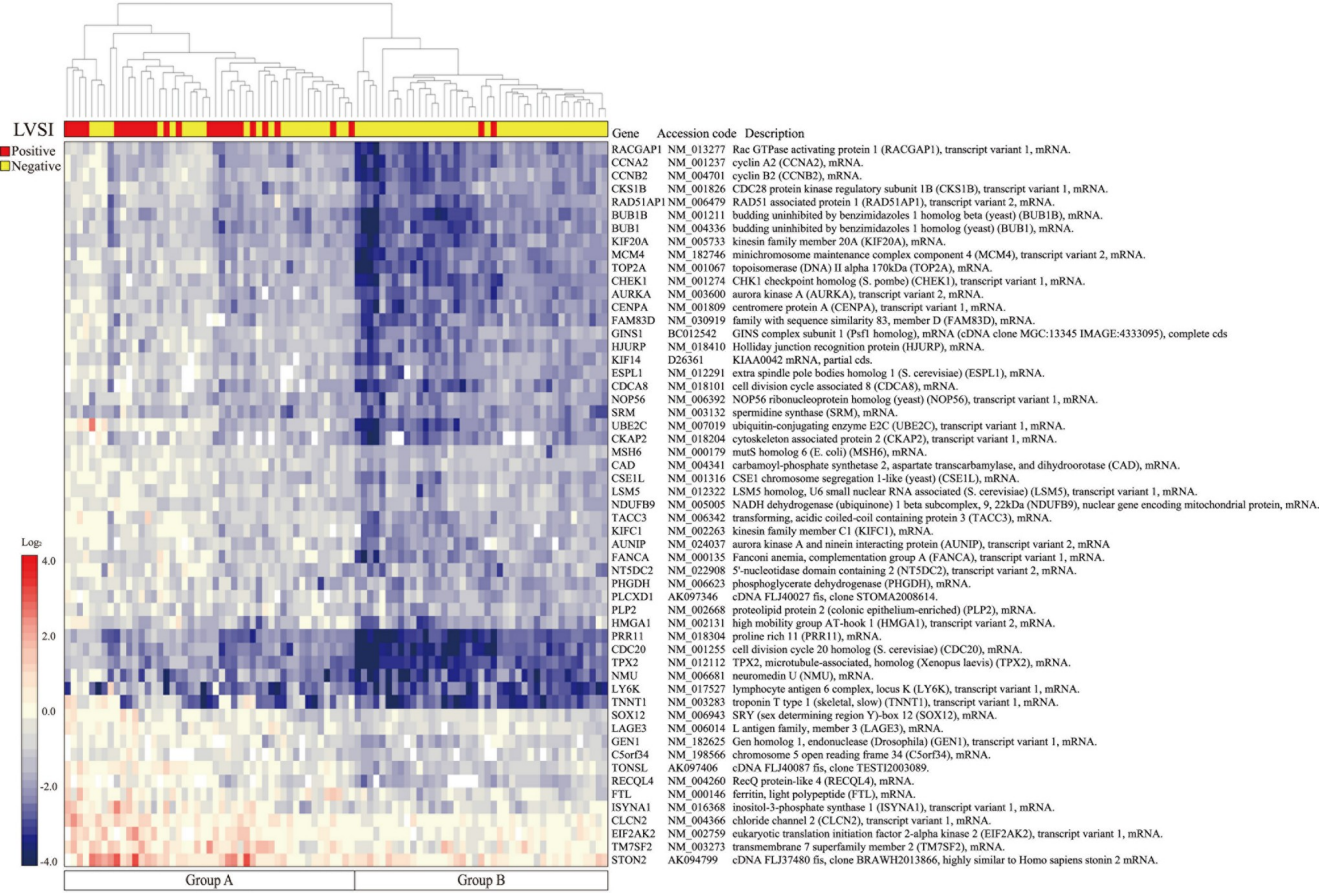
Hierarchical clustering of 55 candidate genes with statistically differentiated expression between patients with and without LVSI. On the heat map, red represents up-regulation and blue represents down-regulation. Two main groups, A and B, were formed.

In order to evaluate the hypothesis that gene expression profiles in the primary EC are able to determine the presence or absence of LVSI, we performed DNA microarray analysis using the 88 EC tumor samples. Finally, 55 candidate genes (hereinafter referred to as a “the 55-gene signature”) that were significantly differentially expressed between LVSI-positive and LVSI-negative samples according to Student’s t test (P < 0.005) were extracted. (Fig 1). Functional category of these genes included cell cycle-related genes (n = 20) and DNA repair-related genes (n = 8).

**Fig 2:**
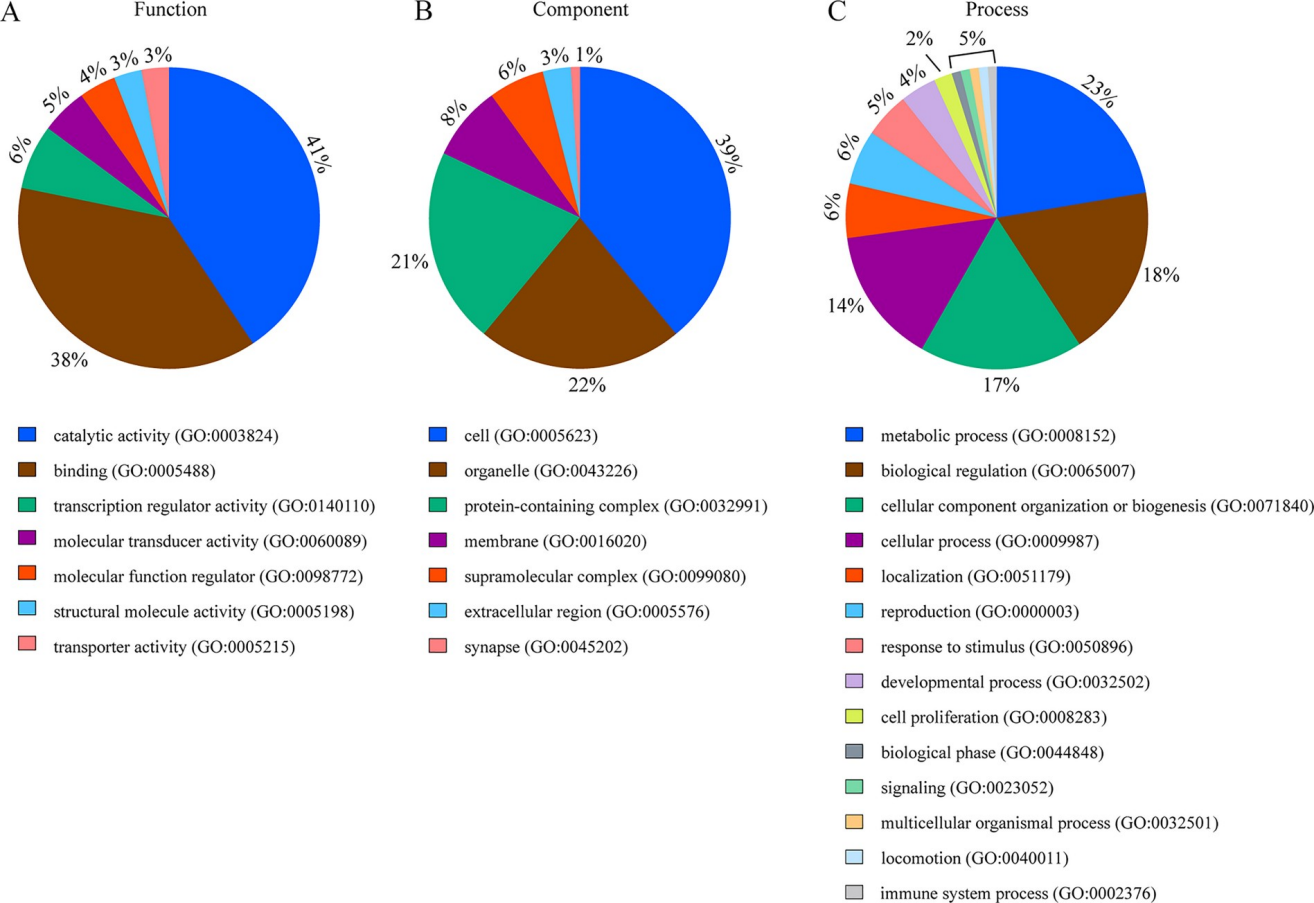
Gene Ontology enrichment analysis of differentially expressed LVSI in endometrial cancer. (A) Molecular function; (B) Cellular component; (C) Biological process.

Next, in order to obtain a system-level view of differential gene expression for LVSI in EC, the 55-gene signature were classified in terms of molecular function, cellular component, and biological process through GO analysis. In terms of molecular function, catalytic activity, binding and transcription regulator activity accounted for 41%, 38%, and 6%, respectively (Fig 2A). As shown in Fig 2B, the major cellular component was cell part (39%), followed by organelle (22%) and protein-containing complex (21%). The top four biological process categories were: (1) metabolic process (23%), (2) biological regulation (18%), (3) cellular component organization or biogenesis (17%), (4) cellular process (14%) (Fig 2C).

### Hierarchical clustering

The 55-gene signature was subjected to hierarchical clustering. All 88 samples were divided into two groups by hierarchical clustering (Fig 1). We evaluated the accuracy of the 55-gene signature. The sensitivity, specificity, positive predictive value, negative predictive value and accuracy were 92%, 63%, 51%, 95%, and 72%, respectively (Table 2).

**Table 2:** Diagnostic performance of the 55-gene signature.

Clustering	LVSI-positive	LVSI-negative	
Group A	24 (TP)	23 (FP)	51.1% (PPV)
Group B	2 (FN)	39 (TN)	95.1% (NPV)
	92.3% (Sensitivity)	62.9% (Specificity)	71.6% (Accuracy)

TP, true positive; FP, false positive; FN, false negative. TN, true negative

PPV, positive predictive value; NPV, negative predictive value; Accuracy = (TN+TP)/(TN+TP+FN+FP)

## Discussion

In the current study, we confirmed that LVSI in EC is strongly associated with poor prognosis. LVSI occurs in about 15% of early-stage patients [13] and is reported to be about 25% when all stages are included [7, 14]. In the present study, the incidence of LVSI in patients with early-stage and all-stage EC were 13.6% (8/59) and 29.5% (26/88), respectively. These findings are similar to the results of previous studies. Our clinicopathological study showed that LVSI was significantly associated with stage, tumor grade, deep myometrial invasion and lymph node metastasis (Table 1). Furthermore, significantly difference in OS was observed patients with and without LVSI (S1 Fig). These data support previous reports showing that LVSI as a predictor of lymph node metastasis and decreased survival in patients with EC [15, 16]. Our data shows that patients with LVSI had significantly lower BMI than those without LVSI. However, Kamal M *et al*. reported that there was no significant association between BMI and LVSI [17]. Some studies have reported a strong association between grade 1 tumor and increased BMI [17, 18]. As LVSI is related to higher tumor grade, lower BMI may indicate a relationship with LVSI.

To develop a diagnostic tool for LVSI in EC, we identified the 55-gene signature that were differentially expressed between LVSI-positive and -negative EC. GO term analysis indicated that LVSI in EC was mainly enriched in multicellular organismal process, biological regulation, cell part, organelle, catalytic activity, and binding. Although 18 genes including ANGPTL4, COL8A1, MMP3, and IL8 were identified to be associated with vascular invasion in a previous study of EC, none of the 55 genes we identified overlapped with the 18 genes [19]. Another study investigated the 214 hepatocellular carcinomas to develop a gene signature that predicts vascular invasion in patients with surgically treated hepatocellular carcinoma [20].

Interestingly, overexpression of the ubiquitin conjugating enzyme E2C (UBE2C) gene was common between our 55-gene signature and 214-gene signature. UBE2C is a member of the anaphase promoting complex/cyclosome and relates the degradation of several target proteins, such as mitotic cyclins and cell cycle progression [21, 22]. Although some studies have reported that the development of LVSI is associated with vascular endothelial growth factor and extracellular matrix related genes [23], in the current study cell cycle-related genes were the most overexpressed genes (20/55, Fig 1). Cell cycle-related genes may also play a role in the development of LVSI.

In the present study, the largest difference in expression level was observed in TNNT1 among the 55-gene signature (data not shown). TNNT1 encodes a protein subunit of troponin, which is involved in the contraction of striated muscle. In the context of cancer, tumor progression of gallbladder carcinoma is positively associated with TNNT1 expression [24]. Recently, it has been reported that TNNT1 mRNA expression is higher in several cancer tissues, such as cervix, colon, lungs, ovaries, and testes, than in normal tissues, and is related to cell migration [25]. As for EC, TNNT1 was differentially expressed when mixed-type endometrial carcinomas were compared to pure low-grade endometrioid adenocarcinoma [26]. Our data suggested that TNNT1 might be important for LVSI development and metastatic spread.

We classified the 88 samples into LVSI positive and negative groups by hierarchical clustering using the gene set consisting of the 55-gene signature. Regarding diagnostic accuracy of the gene set, sensitivity and negative predictive value were both high (92% and 95%, respectively); further, specificity and positive predictive value were both moderate (63% and 71%, respectively) (Table 2). These data showed that most patients in the LVSI-negative group were diagnosed definitely. The 55-gene signature can contribute to pathological diagnosis of LVSI in EC.

Predicting LVSI before surgery is important when deciding a suitable surgical treatment. For example, lymphadenectomy can be omitted in low-risk patients such as those with stage 1A grade 1 EC without LVSI. Currently, however, the presence of LVSI is difficult to predict before surgery and is typically detected at pathological examination of surgically resected specimens. A previous study reported that serum levels of CA125 and fibrinogen before surgery were significantly associated with LVSI [27]. If LVSI is predicted by endometrium biopsy specimen using the 55-gene signature, patients with EC undergoing hysterectomy can decide whether to undergo simultaneous lymphadenectomy.

The current study has some limitations. First of all, the samples analyzed by DNA microarray had heterogeneous histology including not only endometrioid but also non-endometrioid (serous and clear) type. Several papers have reported the molecular heterogeneity of various histologic types of EC [28–30]. We confirmed that the results of hierarchical clustering in only endometrioid type could be similar to that of all histological types of non-endometrioid (data not shown). One reason is that endometrioid type was much more prevalent in our samples (78/88, 88.6%). The other reason is that the influence of histological heterogeneities may be low on LVSI.

Another limitation is that our study conducted only a differential expression analysis between LVSI-positive and LVSI-negative. Yoshida et al. investigated the biomarkers associated with lymph node metastasis for preoperative diagnoses to minimize lymphadenectomy in cases with low-risk of recurrence, and showed SEMA3D mRNA and TACC2 isoforms expressed through a novel promoter to be promising biomarkers using Cap Analysis Gene Expression (CAGE) [31]. The benefits of CAGE are the ability to comprehensively analyze promoter-dependent gene expression, show genetic mutations that cannot be detected with RNA sequencing or microarray, and easily discover the binding motifs of transcription factors. The DNA microarray in our method cannot detect the genetic alterations of the promoter region. If a differential gene expression assay between LVSI-positive and LVSI-negative in our samples was performed using CAGE, novel biomarkers for LVSI could be found.

Finally, the main objective of the current study is to develop a molecular test that could allow prediction of LVSI before hysterectomy. In order for the study to be clinically relevant, we needed to look at the genetic profile of the endometrial biopsy tissue and correlate that with the presence of LVSI on the hysterectomy specimen and the presence of lymph node metastasis. However, the expression of the primary sample is similar to that of endometrial biopsy. Colas E et al. reported that the differential expression of molecular biomarkers in primary endometrial tumors was correlated to their expression levels in corresponding uterine endometrial biopsies [32]. Our data showed that two resulting clusters, groups A and B, were significantly associated with lymph node metastasis (P = 0.008) (data not shown). Two groups separated by hierarchical clustering were able to predict lymph node metastasis beyond GOG33 [33].

In conclusion, LVSI was significantly associated with poor prognostic factors in EC, such as higher tumor grade, lymph node metastasis, deep myometrium invasion, advanced stage and prognosis. The 55 candidate genes selected by DNA microarray had clinically important information that was useful for predicting LVSI in EC. Our study findings suggest that the 55-gene signature can be used as a promising indicator in clinical management of EC and may be also useful for understanding the underlying mechanism of LVSI.

## Supporting information

S1 Fig Overall survival curves for patients with and without LVSI. (TIF)
