# Supplementary figures and images for: Prediction of lymphovascular space invasion in endometrial cancer using the 55-gene signature selected by DNA microarray analysis
Source: PLoS One. 2019 Sep 26;14(9):e0223178. doi: 10.1371/journal.pone.0223178 (PMC6762169; doi:10.1371/journal.pone.0223178)

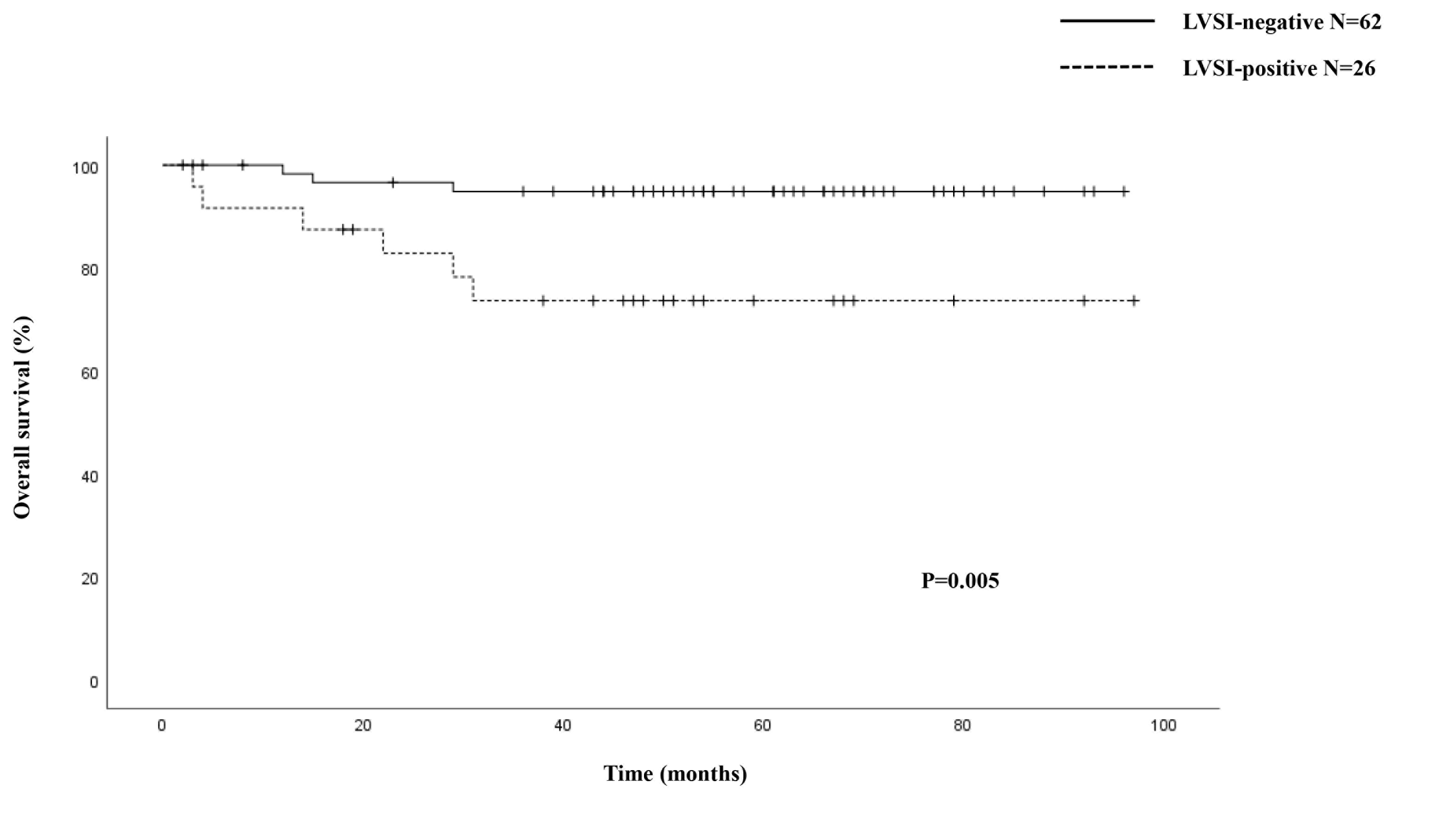

Supplement: S1 Fig — (TIF) [file pone.0223178.s001.tif]
